# ENPEP as a potential predictor of immune checkpoint inhibitor efficacy

**DOI:** 10.1002/cam4.4475

**Published:** 2021-12-03

**Authors:** Aoyun Wang, Han Chu, Zheng Jin, Zhihua Gong, Qingzhu Jia, Bo Zhu

**Affiliations:** ^1^ Institute of Cancer, Xinqiao Hospital Third Military Medical University Chongqing China; ^2^ Chongqing Key Laboratory of Tumor Immunotherapy Chongqing China; ^3^ Center of Growth, Metabolism and aging, Key Laboratory of Bio‐Resources and Eco‐Environment, College of Life Sciences Sichuan University Chengdu China; ^4^ Research Institute GloriousMed Clinical Laboratory (Shanghai) Co., Ltd Shanghai China

**Keywords:** biomarker, ENPEP, immunotherapy, macrophage

## Abstract

**Background:**

The gene ENPEP encodes glutamyl aminopeptidase, which can cut N‐terminal aspartic acid from angiotensin II, and is related to tumorigenesis and immune microenvironment, however, the association between the expression of ENPEP and benefits of immune checkpoint inhibitors (ICIs) has had no investigation.

**Methods:**

We assess the immunotherapeutic predictive performance of ENPEP expression and mutation in multiple cohorts, including one discovery cohort (Pender cohort), four validation cohorts (Hugo cohort; Liu cohort; Mariathasan cohort; Zhao cohort), and one mutation cohort (Miao cohort). Cohorts from The Cancer Genome Atlas (TCGA) were used to explore mechanism and analysis prognosis.

**Results:**

In the discovery cohort, patients with lower ENPEP expression had superior response rates (47.2% vs. 36.1%) and over‐all survival (OS) (HR [95% CI] = 0.61 [0.39–0.96]; *p *= 0.032) compared with those with higher ENPEP expression. The association between ENPEP and immunotherapy efficacy was consistently observed in validation cohorts (Hugo: OS HR [95% CI] = 0.41 [0.11–1.45], *p *= 0.158; Liu: OS HR [95% CI] = 0.73 [0.44–1.20], *p *= 0.211; Mariathasan: OS HR [95% CI] = 0.84 [0.65–1.09], *p *= 0.181; Zhao: OS HR [95% CI] = 0.20 [0.04–1.01], *p* = 0.033; Pooled cohort: OS HR [95% CI] = 0.76 [0.61–0.95], *p *= 0.015), and in the mutation cohort (ENPEP mutation vs. wild type (WT), OS HR [95% CI] = 0.46 [0.26–0.93], *p* = 0.017). Reliably, ENPEP is associated with M2 macrophage infiltration and activation in TCGA.

**Conclusions:**

Our results demonstrated ENPEP is a potential biomarker to classify patients’ response to ICIs treatment.

## Graphical abstract

This study examines the effects the expression of the gene ENPEP may have on predicting performance of immune checkpoint inhibitors in treating various types of cancers. We have found that lower expression of ENPEP leads to a significantly greater response to immune checkpoint inhibitor, along with better overall survival. Thus, we propose ENPEP as a novel biomarker for predicting patients’ response to immune checkpoint inhibitors.

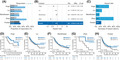

## 1 INTRODUCTION

Immune checkpoint inhibitors (ICIs), which are represented by programmed cell death (ligand) 1 (PD‐1/PD‐L1) inhibitors or cytotoxic T lymphocyte antigen 4 (CTLA‐4) inhibitors, have achieved remarkable success across multiple types of cancers.^1^, ^2^, ^3^, ^4^, ^5^ However, despite improving overall survival compared to other conventional treatments, patients respond to ICIs in a limited subset.^6^, ^7^ Thus, it is important to investigate biomarkers for their response to ICIs, in terms of clinical efficacy and understanding ICI resistance mechanisms.^8^, ^9^

Previous studies have developed various biomarkers to differentiate patients who could benefit from ICI treatment, such as PD‐L1 immunohistochemistry (IHC) of tumor tissue, tumor mutational burden (TMB), tumor‐infiltrating lymphocytes (TILs), and microsatellite instability (MSI) status.^10^, ^11^, ^12^, ^13^ However, the results of PD‐L1 IHC are not completely consistent with patients’ response to ICIs.^14^, ^15^ Also, calculations for TMB lack a standard formula.^16^ Along the same lines, TILs are hardly used in a clinical setting due to their relative high cost, and examination of a patient's MSI status is limited, with spatial heterogeneity of tumors. Therefore, novel and reliable predicting biomarkers are beneficial to the future clinical use of ICIs.

The efficacy of immunotherapy is affected by the tumor microenvironment (TME).^17^ Tumors have complex interactions with their microenvironment, leading them to develop a unique immune response.^18^ The TME is composed of various immune cells, including T cells, natural killer cells, macrophages, neutrophils, and dendritic cells. Immune cells in the TME monitor and prevent cancer cell proliferation and growth.^19^ However, these immune cells play different roles in the TME depending on the type of cancer cells they act upon.^20^ For example, T cells directly kill tumor cells, signifying that successful T cell infiltration is associated with better immunotherapy efficacy.^21^ Neutrophils and tumor‐associated macrophages could prevent other immune cell activation and regulate the immune escape of cancer cells, which may also affect the immunotherapy efficacy.^22^, ^23^ Better understanding of TMEs may help utilize ICIs in clinical practice.

Glutamyl aminopeptidase, encoded by ENPEP, is a membrane protein, which can cut the N‐terminal aspartic acid of angiotensin II, used to increase blood pressure.^24^ In previous studies, ENPEP has been shown to contribute to the development of the immune system. ENPEP has also been associated with tumorigenesis in some cancers, such as breast cancer, leukemia, and renal cancer.^25^, ^26^, ^27^, ^28^ Recently, ENPEP expression levels have been shown to be a prognostic factor in colorectal cancer, linked to the increased survival of patients.^29^ However, the impact of ENPEP on the prognosis of ICIs treatment and the association between ENPEP expression and TME has not been examined.

Here, based on published and supported ICI treatment data, we investigate the relationship between ENPEP expression and the prognosis of ICIs treatment. TCGA database will be used to explore the possible mechanism of unknown ENPEP effects in the TME.

## 2 METHODS

### 2.1 Public data collection

The RNA‐seq data, clinical information, and mutation information of 33 cancer types in the TCGA database (https://cancergenome.nih.gov/) were downloaded using the R package from TCGAbiolinks (https://bioconductor.org/packages/release/bioc/html/TCGAbiolinks.html). RNA‐seq information with sequencing quality “B,” “C,” or “D” were excluded. Considering the limited event cases (event rate <5%), PCPG, PRAD, TGCT, and THCA were excluded from our study. Types HNSC and CESC were excluded owing to their special virus infection environment. LAML and DLBC types were excluded, as they were not solid tumors.

**FIGURE 1:**
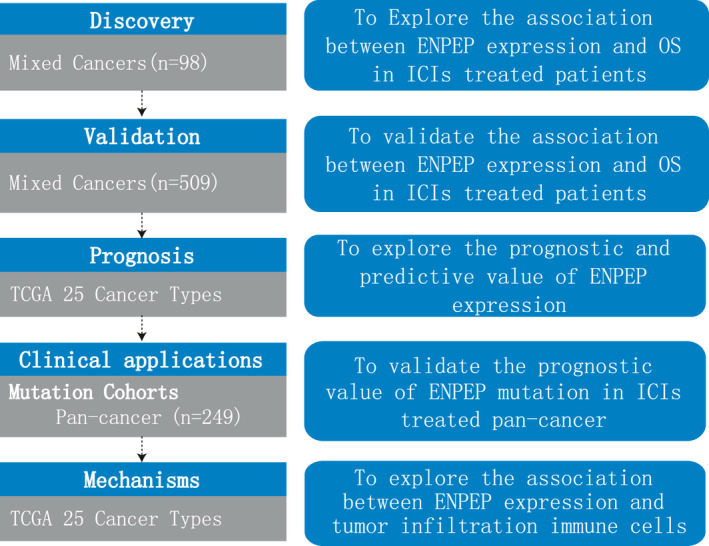
Flow chart of the study. ENPEP, glutamyl aminopeptidase; ICIs, immune checkpoint inhibitors; OS, overall survival; TCGA, The Cancer Genome Atlas

The clinical data and RNA‐seq data of two melanoma cohorts (Hugo cohort and Liu cohort), one bladder cancer cohort (Mariathasan cohort), one GBM cohort (Zhao cohort), and one pan‐cancer cohort (Pender cohort) were collected from published studies.^30^, ^31^, ^32^, ^33^, ^34^ The mutation data and clinical information of another pan‐cancer cohort (Miao cohort) were extracted from the cbioportal database (http://www.cbioportal.org/). All patients had been treated with ICIs (anti‐PD‐1/PD‐L1, anti‐CTLA4, or anti‐PD‐1/PD‐L1 combined with anti‐CTLA4). Response Evaluation Criteria in Solid Tumors (RECIST) version 1.1 was used to evaluate ICIs treatment efficacy. Patients were defined by their response to ICIs treatment when they achieved complete response (CR), partial response (PR) or stable disease (SD) for longer than 6 months. All patients were divided into two groups, low and high, by the median value of their ENPEP expression. The flow diagram of this study was shown in Figure 1.

### 2.2 Single sample gene set enrichment analysis

The immune infiltration scores of M2 macrophage in each sample of TCGA were calculated using Single Sample Gene Set Enrichment analysis (ssGSEA) method in R package “GSVA” (http://bioconductor.org/packages/release/bioc/html/GSVA.html). M2 macrophage signature were collected from a published study.^35^ The results were considered statistically different when *p*‐values were <0.05.

### 2.3 Statistical analysis

OS between different groups was compared by log‐rank test and the Kaplan–Meier (KM) method. Univariate Cox analysis was used to evaluate lower ENPEP expression as the risk (hazard ratio [HR] >1) or protective (0 < HR < 1) for prognosis in ICIs treatment. Chi‐square test was used to compare responder numbers between patients harboring ENPEP mutation and patients without ENPEP mutation. The results achieved statistical difference when *p* < 0.05 in the above analysis. All statistical analyses were performed by the R package 4.0.0.

## 3 RESULTS

### 3.1 ENPEP expression is associated with clinical efficacy of ICIs treatment in the discovery cohort

**FIGURE 2:**
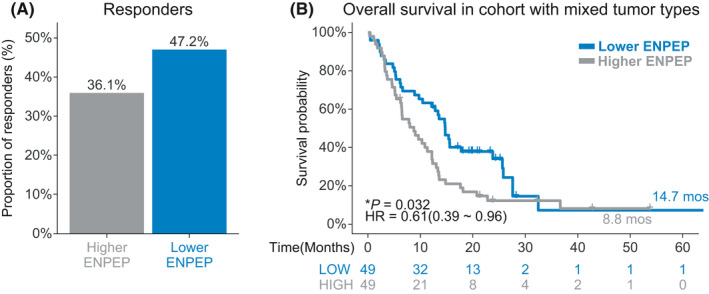
Association between ENPEP expression and ICIs treatment efficacy in discovery cohort. (A) Proportion of responders to ICIs treatment in the high ENPEP expression group or low ENPEP expression group, of the pan‐cancer cohort. (B) Kaplan‐Meier (KM) survival estimate of overall survival (OS) comparing the high ENPEP expression group and low ENPEP expression group, in the pan‐cancer cohort

The discovery cohort consisted of 98 patients with mixed tumor types. The main tumor types of the discovery cohort were lung cancer (26, 26.5%), BRCA (13, 13.2%), and SKCM (11, 11.2%). In the discovery cohort, patients with lower ENPEP expression had a higher response to ICIs treatment rates (47.2% vs. 36.1%, Figure 2A) when compared with patients with higher ENPEP expression. Consistent with response rate, the results of univariate Cox regression show that higher ENPEP expression was a risk factor (HR [95% CI] = 0.61 [0.39–0.96], Figure 2B) for survival. Patients with lower ENPEP expression experienced superior OS (median OS 14.7 vs. 8.8 months, *p *= 0.032, Figure 2B) than patients with higher ENPEP expression. These findings indicated that lower ENPEP expression is associated with better clinical efficacy of ICIs treatment.

### 3.2 ENPEP expression is associated with clinical efficacy of ICI treatment in the validation cohorts

**FIGURE 3:**
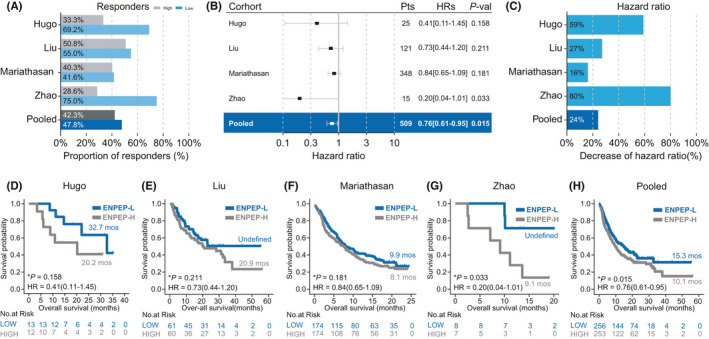
Association between ENPEP expression and ICIs treatment efficacy in validation cohorts. (A) Proportion of responders in high ENPEP expression group or low ENPEP expression group of ICIs treated cohorts. (B) Univariate Cox regression according to ENPEP expression median in ICIs cohorts. (C) Percent reduction of hazard ratio (HR) induced by lower ENPEP expression in ICIs cohorts. (D–H) Kaplan‐Meier (KM) survival curves of overall survival (OS), comparing the high ENPEP expression group and low ENPEP expression group in ICIs treated cohorts

In the validation cohorts, higher response rates (Hugo: 69.2% vs. 33.3%; Liu: 55.0% vs. 50.8%; Mariathasan: 41.6% vs. 40.3%; Zhao: 75% vs. 28.6%; Pooled: 47.8% vs. 42.3%, Figure 3A) were observed in patients with lower ENPEP expression. Lower ENPEP expression was protective (Hugo: HR [95% CI] = 0.41 [0.11–1.45]; Liu: HR [95% CI] = 0.73 [0.44–1.20]; Mariathasan: HR [95% CI] = 0.84 [0.65–1.09]; Zhao: HR [95% CI] = 0.20 [0.04–1.01]; Pooled: HR [95% CI] = 0.76 [0.61–0.95]; Figure 3B,C) for patients who received ICIs treatments. In the Zhao cohort and the pooled cohort, the OS of patients with lower ENPEP expression was significantly longer than the OS of patients with higher ENPEP expression (Zhao: Undefined vs. 9.1 months, *p *= 0.033; pooled: 15.3 vs. 10.1 months, Figure 3G,H). Longer OS was also shown in patients with lower ENPEP expression in the Hugo cohort, Liu cohort, and Mariathasan cohort, but these results were not significant (Hugo: 32.7 vs. 20.2 months, *p *= 0.158; Liu: Undefined vs. 20.9 months, *p *= 0.211; Mariathasan: 9.9 vs. 8.1 months, *p* = 0.181; Figure 3D–F). To avoid statistical bias caused by limited patient numbers of Hugo cohort and Zhao cohort, we pooled Liu cohort and Mariathasan cohort and performed survival analysis (Figure S1). Consistently, Higher response rate and longer OS was found in patients with ENPEP low expression (45.4% vs. 43.2%, 13.4 vs. 9.8 months, *p* = 0.062, HR [95% CI] = 0.80 [0.64–1.01], Figure S1). These findings were consistent with the analysis results of the discovery cohort, indicating that lower ENPEP expression is associated with favorable clinical benefit, through increased response to ICIs treatment, across all four datasets.

### 3.3 ENPEP mutation is associated with clinical efficacy of ICI treatment in pan‐cancer cohort

ENPEP mutation could be a feasible cut‐off value for ICIs in clinical practice. We first investigated the mutation rate of ENPEP in 25 cancers in the TCGA databases (Figure S2). The ENPEP mutation rate in patients of cancer types ACC, BLCA, COAD, ESCA, GBM, LGG, LUAD, LUSC, OV, READ, SKCM, STAD, UCEC, UCS was >1% (Figure S2). Type SKCM patients have the highest ENPEP mutation rate (15.2%; Figure S2). The top five cancers were SKCM (15.2%), UCEC (9.8%), COAD (5.5%), STAD (4.3%), and LUSC (4.1%).

**FIGURE 4:**
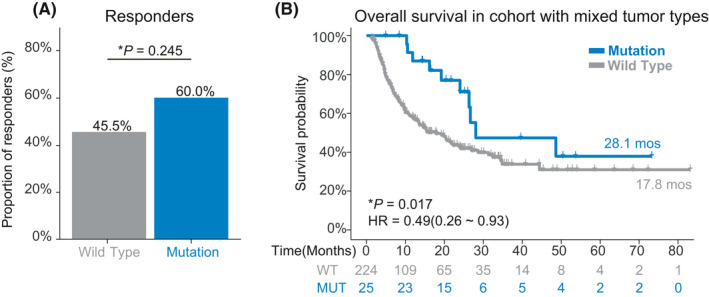
Association between ENPEP mutation and ICIs treatment efficacy in the Pan‐cancer cohort. (A) Proportion of responders in ENPEP missense mutation group or ENPEP wild type group of pan‐cancer cohort. (B) Kaplan‐Meier (KM) survival curves of overall survival (OS), comparing the ENPEP missense mutation group and ENPEP wild type group in the pan‐cancer cohort

We next investigated the effect of ENPEP mutation on prognosis of ICIs treatment in a pan‐cancer cohort (Miao cohort) which was comprised of 249 patients, with more than 10 types of tumors. Melanoma (151, 60.6%), NSCLC (56, 22.5%), and bladder cancer (27, 10.8%) were the main type of tumors in the Miao cohort. 12.4% of patients in the Miao cohort harbored ENPEP mutations, and most ENPEP mutations were missense mutations (80.6%). Patients harboring ENPEP mutations had higher but not significant response rates (60.0% vs. 45.5%, *p* = 0.245, Figure 4A) than wild‐type patients. Superior OS (28.1 vs. 17.8 months, *p *= 0.017, Figure 4B) was observed in patients with ENPEP mutation. And ENPEP mutations were protective for survival (HR [95% CI] = 0.46 [0.26–0.93], Figure 4B). We also found that melanoma patients with ENPEP mutations had longer OS (28.1 vs. 15.5 months; Figure S3) than wild‐type melanoma patients; however, the result was not significant (*p *= 0.064). These findings indicate the association between ENPEP mutation and the better prognosis of ICI treatment.

### 3.4 Lower ENPEP expression was not a prognostic factor in TCGA

We next investigated the influence of lower ENPEP expression on prognosis in the TCGA database. No significant difference was shown in OS between the lower ENPEP expression group and the higher ENPEP expression group in a pooled cohort (pooled cohort: HR [95% CI] = 0.93 [0.85–1.02], *p* = 0.140, Figure S4), and except for LGG, MESO, KIRP, and KIRC, no significant results was observed, indicating that lower ENPEP expression was not influencing the prognosis of non‐ICIs treatment for most cancers.

### 3.5 Potential mechanisms of ENPEP expression predicting the efficacy of ICIs treatment

**FIGURE 5:**
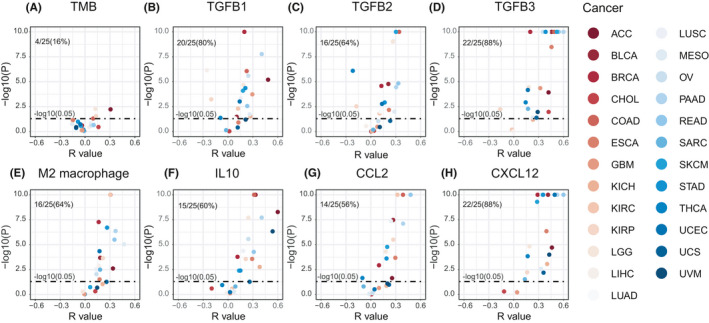
Correlation between ENPEP expression and M2 macrophage in TCGA. (A) ENPEP expression is not correlated with TMB in most cancers. (B) ENPEP expression is positively correlated with M2 macrophage scores in most cancers; M2 macrophage signature scores were calculated by ssGSEA method. (C–H) ENPEP expression is positively correlated with CCL2, CXCL12, IL10, TGFB1, TGFB2, TGFB3 expression in most cancers. All *p* values were estimated by the Pearson correlation test

Next, we examined the association between ENPEP expression and TMEs. Based on the Whole‐exome sequencing (WES) data from TCGA database, we found that ENPEP expression is not correlated with TMB in most cancers (16%, Figure 5A). Previous studies have shown that the expression of ENPEP is regulated by TGF‐β, which is an important factor for the M2 macrophage inhibition of CD8^+^ T cell activation.^36^, ^37^ We found ENPEP is correlated with TGF‐β coding genes TGFB1(80%), TGFB2(64%), TGFB3(88%, Figure 3B–D) in most cancers. Consistently, we found ENPEP expression is positively correlated with M2 macrophage scores in most cancer (64%, Figure 3E). IL10 is another factor secreted by M2 macrophage for CD8^+^ T cell inhibition. The correlation analysis results of IL10 is consist of M2 macrophage and TGF‐β genes (60%, Figure 3F).

To further investigate the possible effects of ENPEP on M2 macrophage, we next investigated the association between ENPEP and macrophage chemokines. We found there is a correlation between ENPEP and CCL2 (56%, Figure 5G), and especially CXCL12 (88%, Figure 5H). Our findings suggested that ENPEP is associated with M2 macrophage infiltration and activation in the TME.

## 4 DISCUSSION

By using collected and supported data, our study has shown that patients with ENPEP mutations or lower ENPEP expression were more likely to respond favorably to checkpoint blockade treatment. ENPEP mutations, and therefore lower ENPEP expression, were associated with longer OS of ICI‐treated patients in pan‐cancers. To our knowledge, our study is first to investigate the possible mechanistic effect of ENPEP on the TME, and the first to provide evidence that ENPEP is a predictive biomarker for successful ICIs treatment in pan‐cancers.

PD‐L1 and TMB have been the major biomarkers to distinguish ICIs treatment responders from non‐responders. The expression of PD‐L1 in tumor cells has been shown to be induced by two major methods: genetic alteration in tumor cells and IFN‐r produced by T cells.^38^ In the TME, IFN‐r expression induced by T cells is the major factor for tumor cell expressing PD‐L1.^39^, ^40^ Thus, higher PD‐L1 expression is associated with higher IFN‐r level, and higher IFN‐r level is accompanied by increased T cell activation.^41^, ^42^ High PD‐L1 expression in tumor cells usually indicates strong CD8^+^ T cell response in the TME.^43^ In previous studies, PD‐L1 expressed by tumor cells has been shown to be associated with better clinical efficacy of ICI treatment in different cancers.^44^, ^45^ However, in recent clinical trials, patients with negative PD‐L1 immunohistochemistry (IHC) showed a positive response to ICI treatment.^46^, ^47^ In some cases, no association has been observed between PD‐L1 expression and ICI clinical efficacy.

Conversely, TMB symbolizes the intrinsic characteristics of tumor cells and is a proxy for immunogenic neoantigens.^48^ In the early stages of cancer, TMB has been associated with the positive prognosis of ICIs treatment.^11^, ^49^, ^50^ However, the calculation of TMB has no unified standard, and TMB cannot offer a clear cut‐off value for ICIs in clinical practice.^51^ Even in recent studies, the association between TMB and the clinical benefits of ICI treatment is controversial.^52^ Compared to PD‐L1, our study found that lower ENPEP expression is associated with a higher response to treatment rate and better clinical prognosis of ICI treatment and that ENPEP expression is independent of PD‐L1 expression (data not shown). Compared to TMB, our study found that patients with ENPEP mutations experienced longer OS and had higher response rates to ICI treatment than patients without ENPEP mutations. ENPEP mutations could provide a clear cut‐off value for ICIs in clinical practice.

Glutamyl aminopeptidase, the production of ENPEP, has been reported that is broadly distributed in tissues.^53^ In previous studies, Glutamyl aminopeptidase was described BP‐1/6C3 antigen, which could be differentiation marker on normal or transformed pre‐B and immature B cells.^54^ The stromal cell of bone marrow or thymus also show strong expression of glutamyl aminopeptidase.^55^, ^56^ Thus, there were suggestions that glutamyl aminopeptidase may function in early B cell and T cell differentiation.^53^ However, glutamyl aminopeptidase‐deficient mice have been proven could generate normal numbers of T and B cells, it is possible that other peptidases compensated the function of glutamyl aminopeptidase.^54^ The function of glutamyl aminopeptidase on immune cell is still unclear, more molecular evidences are needed.

CD8^+^ T cells, which can kill tumor cells directly in the TME, play a crucial role in the antitumor immune response.^57^ PD‐L1 levels have been shown to indirectly reflect the intensity of the CD8^+^ T cell response.^58^ The tumor mutation burden has been shown to be associated with antigen presentation, which eventually leads to CD8^+^ T cell activation.^48^ Our team initially made efforts to investigate the association between CD8^+^ T cell and ENPEP. However, through examination of literature research or data analysis, ENPEP has not shown a direct effect on CD8^+^ T cells activation (data not shown).

On the other hand, our TME analysis results shown the TGF‐β encoding gene is negatively correlated with ENPEP in KIRP, which was inconsistent with other cancers, and may be an indication that the special secretion environment induces different effects.^36^ More molecular studies involving animal models and cell lines are needed. In total, we found that ENPEP expression is positively correlated with M2 macrophage infiltration and activation, which can lead to failed CD8^+^ T cell activation in the TME. The correlation between ENPEP and M2 macrophage may be one possible explanation for ENPEP expression's effect on the prognosis of ICIs treatment.

In summary, our results indicated that ENPEP expression and ENPEP mutation is associated with ICI treatment efficacy in pan‐cancers. Our study provides evidences that ENPEP is a new, reliable, and convenient biomarker for ICIs in clinical practice.

## CONFLICT OF INTEREST

Author Zheng J is employed by GloriousMed Clinical Laboratory (Shanghai) Co., Ltd. The other authors declare that there are no conflicts of interest.

## AUTHOR CONTRIBUTIONS

The concept of the study was conceived by: QingZhu J, Bo Z; Wrote the manuscript: Aoyun W; Analysis data: Aoyun W; Prepared the figure: Aoyun W, Han C, Zheng J, Zhihua G; Supervision: QingZhu J, Bo Z.

## ETHICAL STATEMENT

Not applicable.

## Supporting information

Fig S1

Fig S2

Fig S3

Fig S4

## Data Availability

The datasets used and analyzed during the current study are available from the corresponding author on reasonable request. Miao cohort data were extracted from the cbioportal database (http://www.cbioportal.org/). The mRNA expression profiles of TCGA patients are available in the Genomic Data Commons database (https://portal.gdc.cancer.gov/).
